# The relationship between gender role attitudes and body image concern and quality of life of Balouch women with the mediating role of marital relationship quality: a cross-sectional study using a structural equation model

**DOI:** 10.1186/s12905-026-04363-9

**Published:** 2026-02-28

**Authors:** Mohammad Hossein Kaveh, Mahnaz Didehvar, Masoud Karimi, Mahin Nazari, Seydamalek Dadkhah, Leila Ghahremani

**Affiliations:** 1 Research Center for Health Sciences, Institute of Health, Department of Health Education and Health Promotion, School of Health, Shiraz University of Medical Sciences, Shiraz, Iran; 2 Student Research Committee, Shiraz University of Medical Science, Shiraz, Iran; 3 Department of public health, School of Health, Iranshahr University of Medical Sciences, Iranshahr, Iran; 4 Department of Nursing, School of Medicine, Iranshahr University of Medical Sciences, Iranshahr, Iran

**Keywords:** Quality of life, body image, Interpersonal Relations, gender role, women

## Abstract

**Background:**

Quality of life is a multifaceted concept influenced by various factors. This study examines the relationship between gender role attitudes, body image concerns, and the quality of life among Balouch women, with a focus on the mediating role of marital relationship quality.

**Methods:**

This cross-sectional study used a structural equation approach to analyze data from 297 married Balouch women attending urban healthcare centers. Proportional stratified sampling was conducted from June to September 2023. Data were collected via questionnaires on demographics, gender role attitudes, body image concerns, marital relationship quality, and quality of life. Analysis was performed using descriptive statistics, regression, and structural equations in SPSS24.

**Results:**

The results showed that the marital relationship quality had a significant positive direct impact on the quality of life of Balouch women. Gender role attitude had no direct relationship with quality of life, but it did affect the quality of life by influencing body image concern. Body image concern had a significant negative direct and indirect relationship with the mediating role of marital relationship quality on the quality of life of Balouch women. The results showed that the fit of the final model was good. (χ2 = 1.138، *p* < 0/001، GFI = 0.9، IFI = 1.023، RMSA < 0.001، CFI = 1.00).

**Conclusions:**

The findings of this study provide a comprehensive framework for understanding the multifaceted factors influencing the quality of life among Balouch women. By addressing societal perceptions, enhancing marital relationships, and implementing targeted interventions, it is possible to improve the overall well-being of women in this community.

**Supplementary Information:**

The online version contains supplementary material available at 10.1186/s12905-026-04363-9.

## Introduction

Quality of life is defined as “the standard of health, comfort, and happiness experienced by an individual or group [1]. It is a multidimensional concept that encompasses various aspects of an individual’s life, including physical health, mental well-being, social relationships, and environmental factors [2]. A particular physical object or experience may be perceived differently by different people. This means that a certain quality may involve content, images, and impressions that vary for different people depending on their gender, age, culture, ethnicity, and religion [3].

While quality of life is an essential area of study for all societal groups, particular attention to women’s quality of life is vital due to its significant impact on family health, future generations, and societal well-being [4, 5]. Like other human and social phenomena, quality of life is influenced by numerous factors, including gender role attitudes, body image concerns, and the quality of marital relationships [6].

Gender role refers to the prevailing expectations in a society about the activities and behaviors that men and women can or cannot engage in [7]. Gender roles have a significant impact on women’s quality of life. Traditional gender roles often limit women’s access to education, employment, and healthcare, which can negatively affect their quality of life [8]. In contrast, when women are empowered to participate fully in society, they are more likely to have better health outcomes, higher levels of education, and greater economic opportunities [9]. Feminine norms dictate a set of certain behaviors to women, and in most cultures, they include things such as kindness to children, pleasantness and sacrifice, beauty, and appearance in interpersonal relationships [10]. Yucel et al. showed that gender role attitudes moderate the relationship between working from home and work-family conflict so that traditional attitudes intensify negative effects for women and positive effects for men [11].

Another factor that affects the quality of life is concern about body image [2]. Body image is a multidimensional construct that refers to the perception of a person’s physical appearance, which can be influenced by various factors, including social and cultural norms, media exposure, and personal experiences. It is associated with various mental health issues, including self-esteem, depression, eating disorder symptoms, and anxiety [12]. The mainstream view of women’s body image is that it is a general experience promoted and reinforced by gender-based cultural socialization [13]. Studies show the contradictory effects of body image on women’s quality of life [2, 14].

Another variable that affects women’s quality of life is the quality of marital relationships [15]. The quality of the marital relationship is a multi-dimensional concept that includes various aspects of the couple’s relationship, including compatibility, sexual satisfaction, happiness, cohesion, and commitment. The quality of marital relationships not only makes people live happier, more energetic, and healthier, but also has a major contribution to the health of parents, the education of the next generations, and longevity [16]. Research shows the connection between gender roles, quality of life, satisfaction with married life, and body image [17–19]. The quality of the marital relationship has a significant direct effect on women’s quality of life, and psychological well-being may partially mediate the effects of the marital relationship on women’s quality of life [6]. There is also a positive relationship between physical attractiveness, life satisfaction, and a healthy lifestyle [20].

The fifth goal of the Sustainable Development Goals emphasizes gender equality and the empowerment of all women and girls [21]. However, women from ethnic minority groups often face compounded challenges due to their ethnicity, experiencing a dual layer of inequality compared to both men and women from dominant groups [22]. Balouch women belong to an ethnic minority group in southeastern Iran with distinct cultural traditions, particularly regarding gender roles, family structure, and marital expectations. In traditional Balouch culture, patriarchal norms are more strongly emphasized, and women often face stricter social expectations regarding modesty, obedience, and body image than in other Iranian ethnic groups [23]. Regional differences in culture and tradition influence attitudes toward gender roles, body image concerns, and the nature of marital relationships, all of which directly affect women’s overall well-being [24, 25]. 

**Fig. 1:**
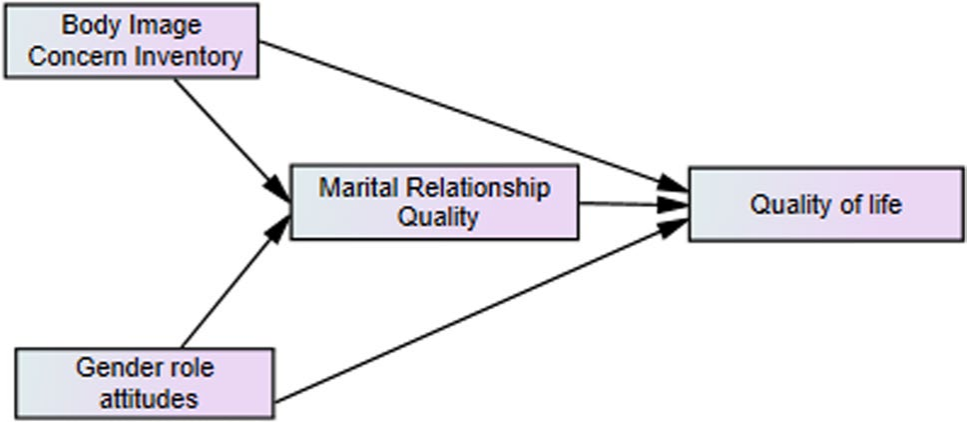
Conceptual model of the study

Understanding the specific experiences of Balouch women can reveal region-specific challenges and opportunities, contributing to more effective, culturally sensitive interventions. Given the lack of research examining the interplay between gender role attitudes, body image concern, marital relationship quality, and women’s quality of life in this context, the present study aims to investigate these relationships among married Balouch women in the city of Iranshahr. The research hypotheses are as follows:

- H1: The quality of marital relationships has a significant positive direct influence on the quality of life of Balouch women.
- H2: Gender role attitudes have a significant positive direct and indirect influence on Balouch women's quality of life, with the mediating role of the quality of marital relationships.
- H3: Body image concern has a significant negative direct and indirect impact on the quality of life of Balouch women with quality of marital relationships playing a mediating role. Figure 1 shows the default model of the study.

## Methods

The present study is a descriptive correlational study conducted from June to September 2023 using the structural equation approach. The statistical population consisted of married Balouch women who referred to the Centers for Integrated City Healthcare Services in the city of Iranshahr, Iran.

Generally, in the structural equation modeling method, the sample size can be set between 5 and 15 observations for each variable measured [26]. Considering that the questionnaire on gender role attitudes in this study contains more questions than other questionnaires and consists of 45 questions, the minimum sample size was estimated to be 225. However, to achieve better generalizability, we considered 305 samples.

for prevention of sampling bias, sampling was done using the proportional stratified method (based on which the population of each center is distributed proportionally). Considering the number of married Balouch women recorded by each center and based on the Sib system, the number of samples for each center was determined. Then, in each center, the list of women was extracted from the system based on the input criteria, and the sampling was done by systematic random sampling. After the researcher selected the samples, he explained the desired project to them in a telephone call, and if they were willing to participate in the project, they were invited to come in person to fill out the questionnaire. A written informed consent form was obtained before the questionnaire was completed. If someone was not willing to take part in the project, the next number on the list was selected.

### Inclusion and exclusion criteria

**Fig. 2:**
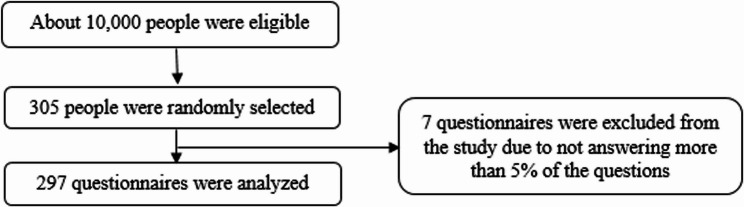
Flowchart of the selection of participants

The criteria for participation in the study were age of childbearing potential (15–49 years), married women, Balouch ethnicity, no mental illness, and no pregnancy, ability to read and write, and full consent to participate in the study. Exclusion criterion was lack of response to more than 5% of the questions in the questionnaire. The flowchart of the selection of participants is shown in Fig. 2.

### The data collection tools

The questionnaire consisted of five parts. The first part of the demographic profile included information about age, level of education, occupation, wife’s income, and husband’s income. The second part of the questionnaire on gender role attitudes was compiled and standardized by Keiani in Iran in 2007. This questionnaire consists of 45 statements rated on a 5-point Likert scale from “strongly agree” to “strongly disagree”. Some questions are reverse scored. In the direct assessment, “I fully agree” with a score of 1 and “I strongly disagree” with a score of 5. The minimum score is 89 and the maximum score is 173. The internal consistency was 0.81, and the retest reliability was 0.77 after two weeks [27]. Cronbach’s alpha coefficient of this questionnaire in the studied population was 0.81.

Body Image Concern Scale contains 19 items that examine a person’s dissatisfaction and concerns about their appearance. This questionnaire was created by Littleton et al. in 2005 and given to 1403 women aged between 18 and 55 [28]. It is scored on a Likert scale. The total score of the questionnaire ranges from 19 to 95, with a higher score indicating a high level of dissatisfaction with the person’s body image or appearance. The validity of this questionnaire was tested using the internal consistency method and the Cronbach’s alpha coefficient was 0.93. The correlation coefficient of the individual questions with the total score of the questionnaire was between 0.32and 0.73 with an average of 0.62 [29]. Cronbach’s alpha coefficient of this questionnaire in the studied population was 0.83.

The revised form of the marital quality scale with 14 questions was created by Busby, Curran, Larsen, and Christensen (1995). It isscored using a 6-point spectrum from (0, we always disagree and 5, we always agree). This instrument consists of three subscales: agreement (items 1–6), satisfaction (items 7–10), and cohesion (items 11–14), which indicate the overall level of marital quality. The Cronbach’s alpha coefficient was estimated 0.92 [30, 31]. Cronbach’s alpha coefficient was 0.91.

The quality-of-life questionnaire is a shortened form of the 36-question quality of life questionnaire. This version was designed by Ware in 1996 [32]. This questionnaire has 8 subscales. Due to the small number of items, the person’s total score is often used. The questions are to be answered with a multiple-choice Likert scale and yes and no responses. The total score of the questionnaire is calculated from the sum of the scores for each question. The minimum and maximum scores are 12 and 48, respectively. Depending on the score they receive based on the questionnaire, people are divided into three categories: weak [12–24], average [25–36], and good [33, 37–48]. Cronbach’s alpha coefficient of this questionnaire in the studied population was 0.83.

### Statistical analyses

Descriptive tests, including frequency, mean and standard deviation, were used to compare the groups using the independent t-test and analysis of variance. Structural equation modeling with AMOS24 software was used to examine the direct and indirect relationships of the variables with quality of life. Descriptive statistics, including the frequency distribution of participants about demographic variables and mean and standard deviation of the values of the research constructs, as well as the Pearson correlation coefficients between the model constructs and the regression were analyzed using SPSS26 software. In this context, structural equation modeling (SEM) was also used with AMOS 26 software. SEM is a powerful method for assessing the relationship between the observed and latent variables in a study. One of the valuable aspects of this method is the simultaneous analysis and processing of the relationship between the variables in the model [34].

The fit indices were Chi-Square/degrees of freedom ratio (X2/df), root mean square error of approximation (RMSEA), goodness of fit index (GFI), comparative fit index (CFI), and incremental fit. The IFI index was used to measure the fit of the final model. C2/df ratio values of 5 or smaller were considered as good fit, RMSEA values smaller than 0.08 were acceptable fit and less than 0.05, and GFI values greater than 0.8 or 0.9 were considered as good fit Finally, CFI and IFI values greater than 0.9 were considered a good fit [35].

### Finding

In this study, 297 people took part. Their meanage was 29.91 ± 7.17 years. The youngest and oldest participants were 17 and 50 years old, respectively. Table 1 shows the frequency distribution of participants based on demographic characteristics.

**Table 1:** Frequency distributions of the participants’ demographic characteristics

variable		*N*	percent
Age	< 25	98	33
	25 to 35	122	41.1
	35–49	77	25.9
	Total	297	100
Education level )participants)	< diplom	74	24.9
	diplom	90	30.3
	> diplom	133	44.8
	Total	297	100
Education level(husbands)	< diplom	80	26.9
	diplom	96	32.3
	> diplom	120	40.4
	Total	296	99.7
Income(participants(	No income	177	59.6
	> 5 million toman*	17	5.7
	5 to 10 million toman	73	24.6
	< 10 million toman	30	10.1
	Total	297	100
Income(husbands)	> 5 mil	90	30.3
	5 to 10 mil	137	46.1
	< 10 mil	69	23.2
	total	296	99.7
Job(participants)	Employed	98	33
	Unemployed	199	67
	Total	297	100
Job(husbands)	Employed	287	96.6
	Unemployed	10	3.4
	Total	297	100

* Each one million tomans is equivalent to 20 dollars now

Table 2 shows the mean values, range, and correlation coefficients of the study variables; also, it depicts the direct and indirect effects of the variables on body image concerns, marital relationship quality, and gender role attitudes on quality of life.

**Table 2:** Descriptive statistics, Pearson correlation coefficients between variables, and direct and indirect effect of variables on quality of life

	variable	Mean(SD)	Possible rang	1	2	3	4	effect of variables on quality of life		
								Direct effect	Indirect effect	Total effect
1	Quality of life	33.69(6.63)	12–48	1	---	----	---	---	---	---
2	Gender role attitudes	144.57(19.04)	45–225	0.01	1	---	---	---	0.05	0.05
3	Body Image Concern Inventory	34.27(13.36)	19–95	− 0.23^**^	− 0.20^**^	1	--	0.26	---	0.26
4	Marital Relationship Quality	51.17(12.30)	0–70	0.30^**^	0.05	− 0.25^**^	1	0.14	----	0.14

** Correlation is significant at the 0.01 level (2-tailed). *p* < 0.001

The findings shown in Table 2 reveal that the quality of life of Balouch women is average. Attitudes towards gender roles, concern about body image, and quality of marital relationships all affect the quality of life of Balouch women. However, these effects are direct and indirect. Attitude toward gender roles has an indirect relationship with the quality of life. This variable can affect quality of life through influencing other variables, such as body image concern. Body image concern has a direct relationship with quality of life and can directly affect quality of life. Marital relationship quality has a direct relationship to quality of life.

**Fig. 3:**
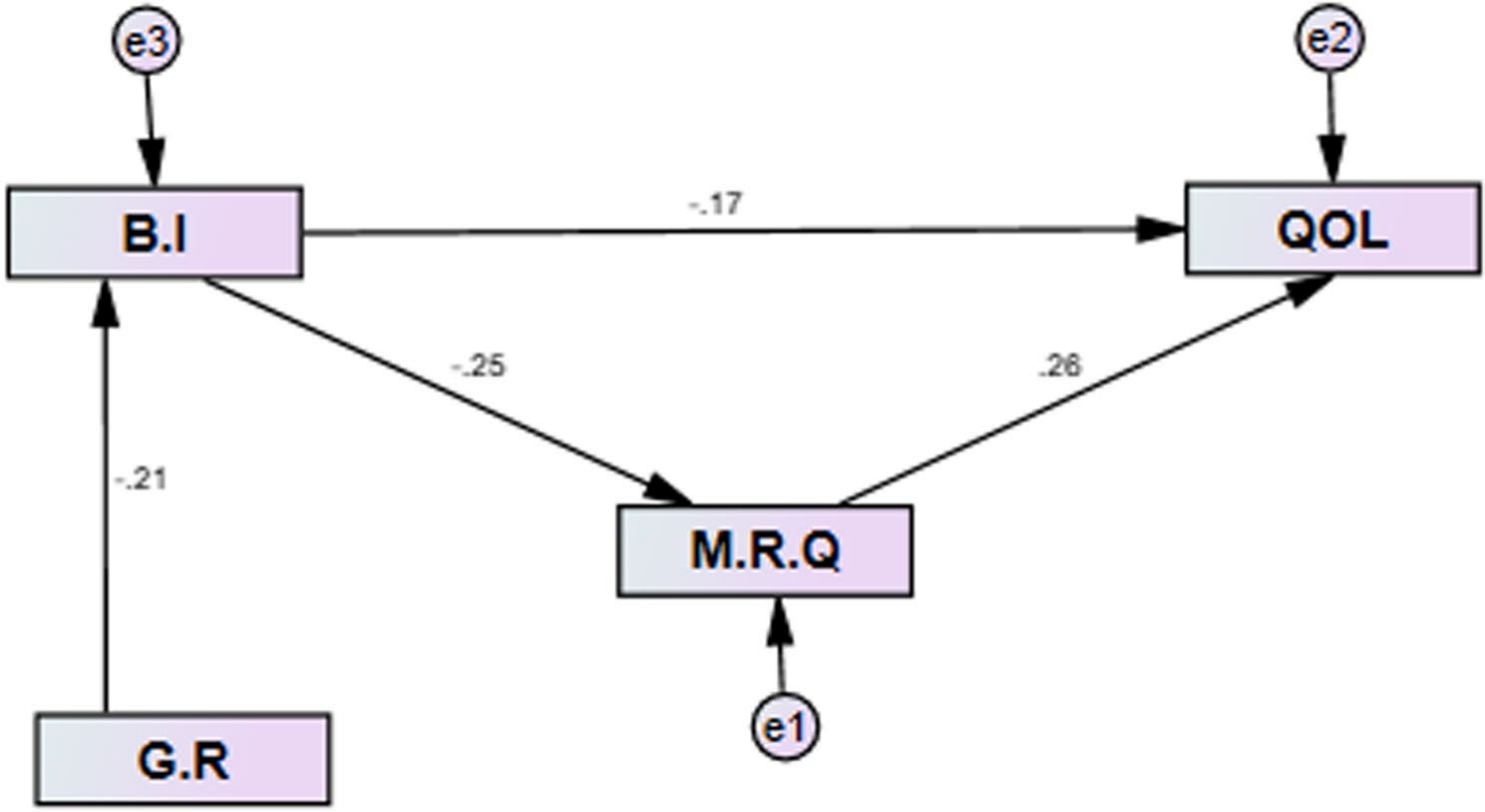
The final model derived from the study. B.I: Body Image Concern Inventory. G.R: Gender role attitudes. M.R.Q: Marital Relationship Quality. QOL: Quality of life

The final model from the SEM analysis (Fig. 3) shows that body image concerns and marital relationship quality are directly related to Balouch women’s quality of life and that gender role attitudes affect quality of life through the variable of body image concerns.

Multiple linear regression analysis shows that body image concern (β=-0.179, *p* = 0.002) and marital relationship quality (β = 0.263, *p* < 0.001) are significant predictors of Balouch women’s quality of life. It is worth noting that the 12% model can predict women’s quality of life (Table 3).

**Table 3:** Multiple linear regression analysis of variables affecting quality of life

Variables	Unstandardized coefficients		Standardβ	t	sig
	B	SE			
Constant	31.18	3.661	---	8.51	< 0.001
Gender role attitudes	-0.01	0.02	-0.03	-0.60	0.54
Body Image Concern Inventory	-0.08	0.02	-0.17	-3.09	0.002
Marital Relationship Quality	0.14	0.03	0.26	4.64	< 0.001

Adjusted R^2^ = 0.12 *p* < 0.001

The findings in Table 3 show that an inverse relationship exists between the attitude toward gender role and concern aboutbody image with the quality of life of women; that is, with the increase of these two variables, the quality of life decreases. There is a positive and direct relationship between the quality of marital relations and the quality of life of women.

Table 4 shows the model fit indices and the acceptable values for each index. The results show that the model fit is acceptable.

**Table 4:** Fit indexes of the model

INdex	Compute index	Accepable range
Chi-Square/Degrees Of Freedom Ratio (X2 /df)	1.138/2	< 5
Goodness-Of-Fit Index (GFI)	0.9	> 0.9
Comparative Fit Index (CFI)	1.0	> 0.9
Incremental Fit Index (IFI)	1.02	> 0.9
Mean Square Error of Approximation (RMSEA)	< 0.001	< 0.080

## Discussion

This study aimed to examine gender role attitudes and body image concerns on the quality of life of Balouch women, with the quality of marital relationship playing a mediating role. Quality of life is a multidimensional concept and needs to be measured from different angles and dimensions. These dimensions are the main components of quality-of-life research [36].

According to the first hypothesis of this research, the results of our study have shown that the quality of marital relationships has a significant direct and positive impact on women’s quality of life. The quality of marital relationships is an intra-individual phenomenon and an individual perception of the spouse and the relationship. Such a definition indicates that marital relationship quality is a unidimensional concept and expresses an individual’s overall evaluation of the spouse and the relationship [37]. Studies consistent with our research have shown a direct and positive relationship between the quality of the marital relationship and quality of life [38–40].

Previous studies have shown that quality of life is significantly influenced by marital relationship patterns [41, 42].

Due to traditional culture, most Iranian women play their role as wives in the sense of “helping their husbands and raising children”, which is beneficial for marital relations and contributes to improvement of their quality of life [43].

In contrast to our second hypothesis, gender role attitudes did not have a direct relationship with the quality of life of Balouch women. Rather, they had an indirect relationship with women’s quality of life by affecting body image. The study by Kayani et al. found a significant negative correlation between gender role attitudes and life satisfaction in working women and men [27].

Some studies have shown that gender role attitudes have a positive relationship with quality of life [44, 45]. The degree of acceptance of gender stereotypes is the degree of a person’s belief in some stereotypical ideas about behavioral characteristics and intellectual qualities attributed to men or women in society [46].

As a result of her research, Audette found that promoting gender equality leads to greater psychological well-being. Furthermore, measures to promote gender equality tend to improve the quality of life for everyone, not just the direct beneficiaries of these measures (women). Men also experience a strong and significant increase in life satisfaction when the sexes are equal [47].

The pace of resistance to stereotypes and schemas in traditional societies like Balouch is slow despite the existence of powerful structures and institutions like religion and cultural beliefs. Today, access to communication technology and media has increased this speed [48].

Our results show that positive gender role attitudes decrease body image concerns, and decreased body image concerns increase women’s quality of life. In contrast to our results, Gillen et al. found a stronger association between more traditional beliefs about “appropriate” gender behavior in social interactions and a stronger orientation toward appearance. Attitudes about the roles of men and women in society may cause women who prefer gendered behaviors to spend more time thinking about and investing in their appearance to meet society’s cultural expectations of women. They may also be sensitive to violations of gender role norms by other women because these violations represent deviations from culturally appropriate behaviors, styles, and attitudes for women [49]. 

The strong differentiation of gender roles in Hispanic/Latino and Asian-American cultures suggests that the body image ideals of the dominant culture are imitated. In the so-called Hispanic cultures of the United States, there seems to be a greater acceptance of larger body sizes that contradict the dominant American ideals, making acculturation an important factor to consider. Social and economic status should also be considered. Women of lower socioeconomic status are less likely to purchase commercial products that support the ideal image [50].

According to our research findings, women with traditional attitudes towards marriage (e.g. men should make important family decisions) may support the traditional belief that women should be altruistic. Prioritizing the needs of others may cause women to expend less energy and worry less about their appearance [49]. 

The results of our research showed that there was a significant negative association between body image concerns and quality of life among Balouch women, which is consistent with the study by Kim et al. The results of their study showed that there was a relationship between physical attractiveness, life satisfaction, and a health-promoting lifestyle. After taking into account age, gender, marital status, education, and employment status, there was a positive correlation [19].

Dissatisfaction with one’s own body and diet is one of the risk factors for the development of eating disorders, which pose a serious threat to women’s emotional and physical health [51]. The research findings of Wilson et al. also showed that body image dissatisfaction in women affected their psychological and social health and ultimately reduced their quality of life [52]. In the study by Tian et al., a high BMI from childhood to middle adulthood was only moderately associated with psychosocial health [53].

The results of the present study showed that body image concerns affected the quality of life of Balouch women by mediating the quality of marital relationships. This means that a reduction in body image concerns increases the quality of marital relationships and ultimately affects women’s quality of life. The results of some studies confirm this finding that the state of body image affects the quality of marital life [54, 55] .

In Hochgraf et al.’s study, the two-way interaction between partners’ perceptions of their spouse’s weight showed that husbands’ perceptions that their spouses were overweight predicted a decrease in spouses’ marital satisfaction and an increase in spouses’ reports of marital conflict during a year. In contrast, the wife’s perception of the husband’s weight was unrelated to changes in marital satisfaction or the husband’s conflict. The results show that concern about one’s weight and that of one’s spouse has negative consequences for marital relationships [56].

## Conclusion

This study showed the relationship between body image concerns and gender role attitudes with the mediating role of marital relationship quality on Balouch women’s quality of life. The fit of the final model was adequate. The results of this study suggest that body image concerns and gender role attitudes play an important role in the quality of life of Balouch women. The quality of marital relationships and concern about body image are directly related to women’s quality of life, i.e. the greater the concern about body image, the lower the women’s quality of life and the higher the quality of marital relationships, the higher the quality of life of the Balouch women.

Therefore, these findings can be used to develop educational programs and psychological interventions. These programs can help Balouch women improve their quality of life by changing their attitudes toward gender roles, reducing concerns about their body image, and improving the quality of their marital relationships.

In addition, these findings can help psychologists, counselors, and policymakers develop and implement intervention programs and policies to improve the quality of life of Balouch women. These could include educational programs to change attitudes about gender roles, intervention programs to address body image concerns, and support programs to improve the quality of marital relationships.

Finally, this research can be an important step towards improving the quality of life of Balouch women. However, there is a need for further research to investigate these issues in more depth and detail. This may include examining the impact of other psychological and social factors on the quality of life of Balouch women and evaluating the effectiveness of the proposed intervention programs.

### Strengths and limitations

One of the strengths of our study is the examination of women’s quality of life and the mediating role of the quality of marital relationships in each ethnic group. To the best of our knowledge, no study on this topic has been conducted before. One of the limitations of our study could be its relatively small sample size. Due to the multi-part questionnaire with many questions, it was challenging for the participants to answer all the questions, and some questionnaires were excluded from the study for this reason. Another limitation of our study was the lack of inclusion of illiterate women. The results of the present study may not be generalizable to women of other ethnicities as it was conducted in a specific ethnic group. Because very few studies have been conducted on Balouch women, it was not possible to compare other cultures with the culture of Balouch.

It is suggested that similar studies should be conducted on illiterate women, women from other tribes with a larger sample size, and also on couples.

## Supplementary Information

Supplementary Material 1.

Supplementary Material 2.

## Data Availability

No datasets were generated or analysed during the current study.

## Abbreviations

B.I.: Body image concern inventory
G.R.: Gender role attitudes
M.R.Q: Marital relationship quality
QOL: Quality of life
SEM: Structural equation modeling
