# Supplementary material for: The relationship between gender role attitudes and body image concern and quality of life of Balouch women with the mediating role of marital relationship quality: a cross-sectional study using a structural equation model
Source: BMC Womens Health. 2026 Feb 28;26:191. doi: 10.1186/s12905-026-04363-9 (PMC13059182; doi:10.1186/s12905-026-04363-9)
Supplement: Supplementary file 1 — Supplementary Material 1. [file 12905_2026_4363_MOESM1_ESM.pdf]

دانشکده های بهداشت، تغذیه و علوم غذایی- دانشگاه علوم پزشکی شیراز

مصوبه کارگروه/کمیته اخلاق در پژوهش

**شناسه:**	IR.SUMS.SCHEANUT.REC.1402.020	
**محل بررسی:**	کارگروه/ کمیته اخلاق در پژوهش دانشکده های بهداشت، تغذیه و علوم غذایی- دانشگاه علوم پزشکی شیراز	تاریخ تصویب: ۱۴۰۲/۱/۲۰
**تصمیم صادره:**	مصوب	
**مصوبه کمیته تخصصی:**	پروپوزال طرح تحقیقاتی/پایان نامه/گزارش مورد، مورد تصویب کارگروه/ کمیته‌ی اخلاق در پژوهش قرار گرفت.<br>توجه:<br>۱. علی‌رغم تصویب مطالعه در کارگروه/کمیته اخلاق در پژوهش، تمامی مسئولیت‌های حقوقی و حرفه‌ای اجرای طرح به عهده مجری ارشد و همکاران مطالعه می‌باشد.<br>۲. تمامی مستندات مطروحه در این مصوبه، بر اساس موارد دریافت شده در تاریخ ۱۴۰۲/۱/۲۰ می‌باشد و ضروری است هرگونه تغییرات و اصلاحات اعمال شده در این مستندات، توسط مجری محترم مطالعه فورا به کارگروه/ کمیته اخلاق مربوطه اطلاع داده شود.	
**عنوان طرح تحقیقاتی (فارسی):**	بررسی رابطه بین نگرش نقش جنسیتی و نگرانی از تصویر بدنی با نقش میانجی کیفیت زندگی زنان و کیفیت روابط زناشویی در شهرستان ایرانشهر، سال 1402	
**عنوان طرح تحقیقاتی (انگلیسی):**	Investigating the relationship between gender role attitudes and body image concerns with women's quality of life and the mediating role of marital relationship quality in Iranshahr city, year 2023	
**مشخصات محقق اصلی:**	نام و نام خانوادگی: دکتر لیلا قهرمانی /ارتقاء سلامت<br>آدرس الکترونیک: ghahramanl@sums.ac.ir	

دکتر ابوالفضل اژدرپور
رئیس کمیته
دانشکده های بهداشت، تغذیه و علوم غذایی- دانشگاه علوم پزشکی شیراز

دکتر ابوذر سلطانی
دبیر کمیته
دانشکده های بهداشت، تغذیه و علوم غذایی- دانشگاه علوم پزشکی شیراز
